# Supplementary figures and images for: TAMC: A deep-learning approach to predict motif-centric transcriptional factor binding activity based on ATAC-seq profile
Source: PLoS Comput Biol. 2022 Sep 12;18(9):e1009921. doi: 10.1371/journal.pcbi.1009921 (PMC9499209; doi:10.1371/journal.pcbi.1009921)

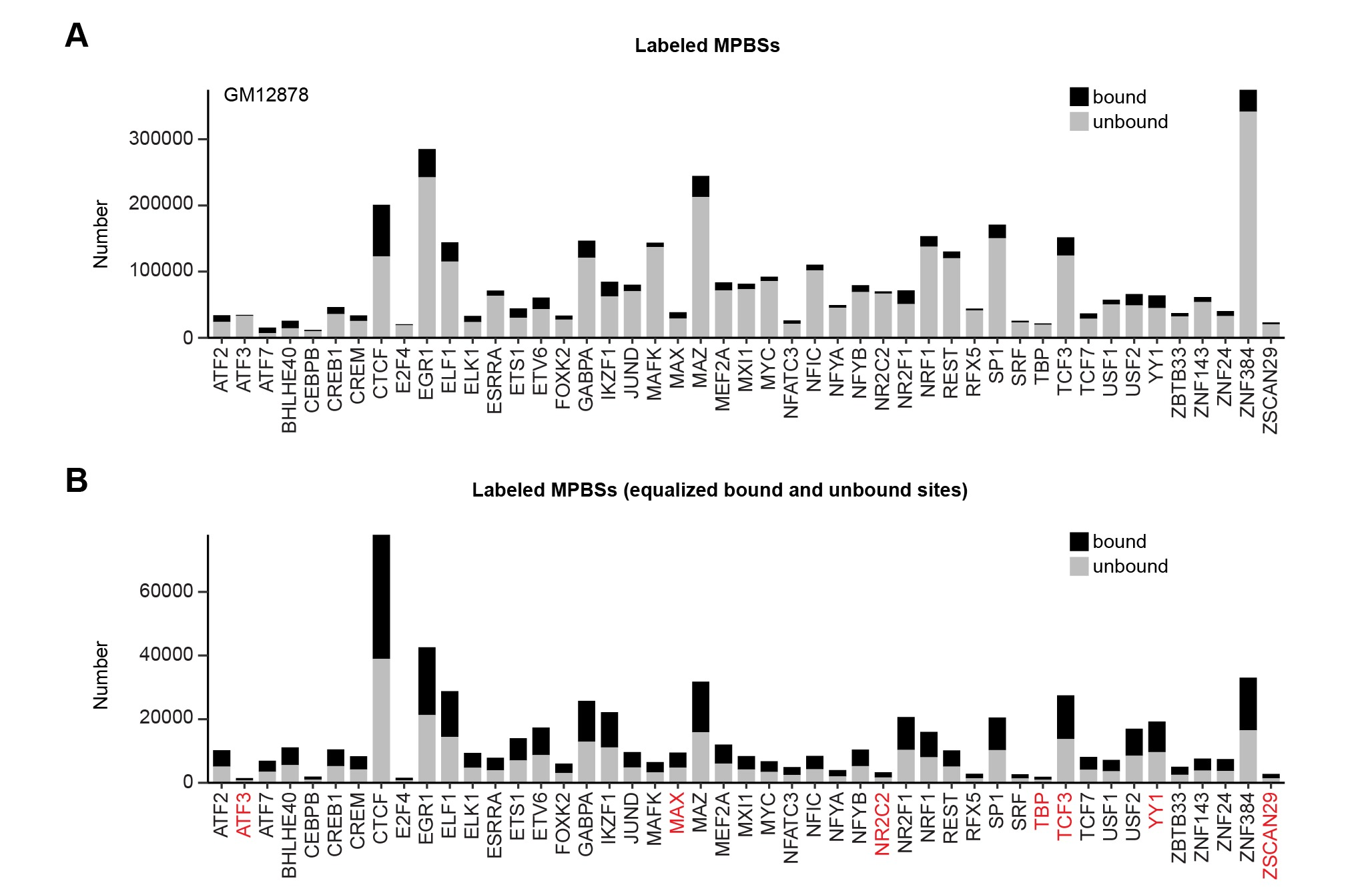

Supplement: S1 Fig — Number of labeled MPBSs for 47 TFs before (A) and after (B) equalization of bound and unbound sites in GM12878 cells (supplementary to Figs 1 and 2). TFs did not show better intra-data classification using TAMC than TOBIAS or HINT-ATAC were highlighted in red. (TIF) [file pcbi.1009921.s001.tif]

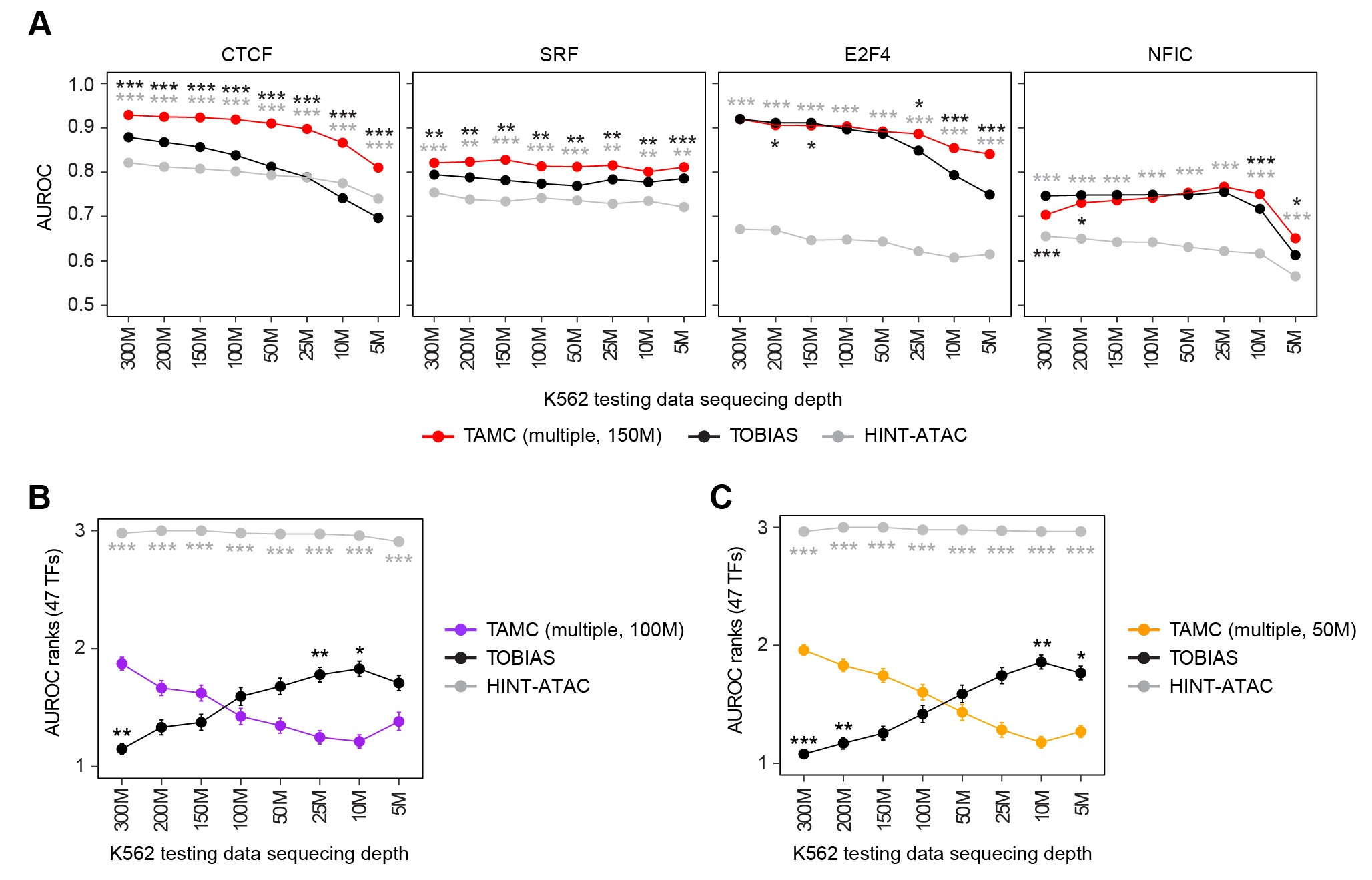

Supplement: S2 Fig — (A) Line graphs compare CTCF, SRF, E2F4 and NFIC binding site prediction performance using trained TAMC model with TOBIAS and HINT-ATAC. The points represent for mean AUROC ± SEM (n = 3 experimental replicates). P-values were calculated using two-sided unpaired Student’s t-test. (B-C) Line graphs compare average cross-data classification performance of TAMC models trained on multiple (GM12878 and hepG2) ATAC-seq data with 100M (A) and 50M (B) sequencing depth with TOBIAS and HINT-ATAC. The performance of models within each line graph were ranked from 1 to 3 for each TF. The higher AUROC is, the lower rank number is given. The points show the average AUROC ranks for 47 TFs for each method and the error bars represent SEM. P-values were calculated using Friedman-Nemenyi test. The cell type and sequencing depth of ATAC-seq used for train TAMC models are indicated within the parenthesis. HINT-ATAC models pre-trained on GM128782 ATAC-seq data were used for all testing, while TOBIAS does not require model training before testing. M denotes million high-quality non-mitochondrial aligned reads. Complete statistic test results and raw AUROC data for all figures are provided in S3 and S4 Tables, respectively. * p < 0.05, ** p < 0.01, *** p < 0.001. (TIF) [file pcbi.1009921.s002.tif]

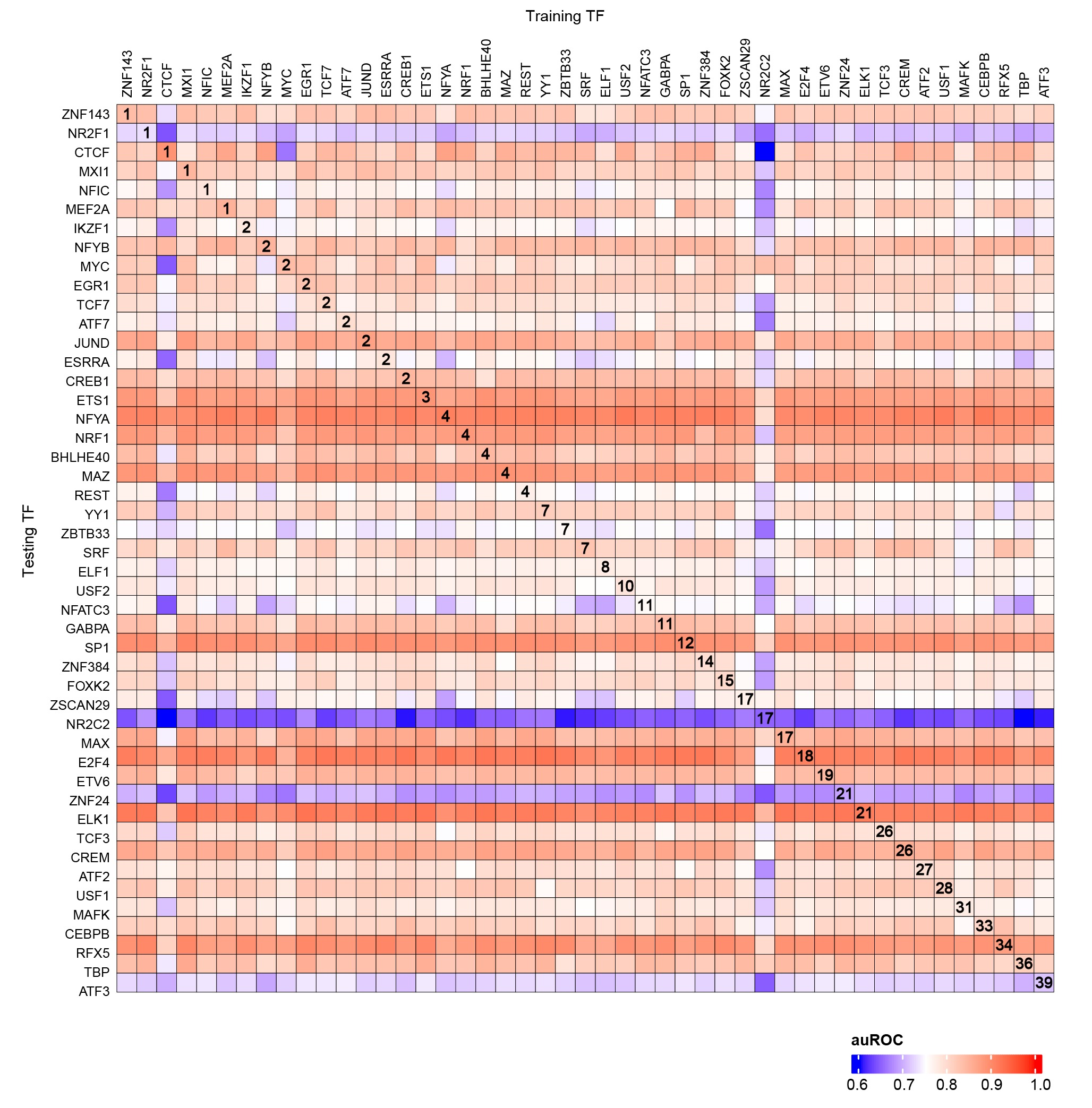

Supplement: S3 Fig — (TIF) [file pcbi.1009921.s003.tif]

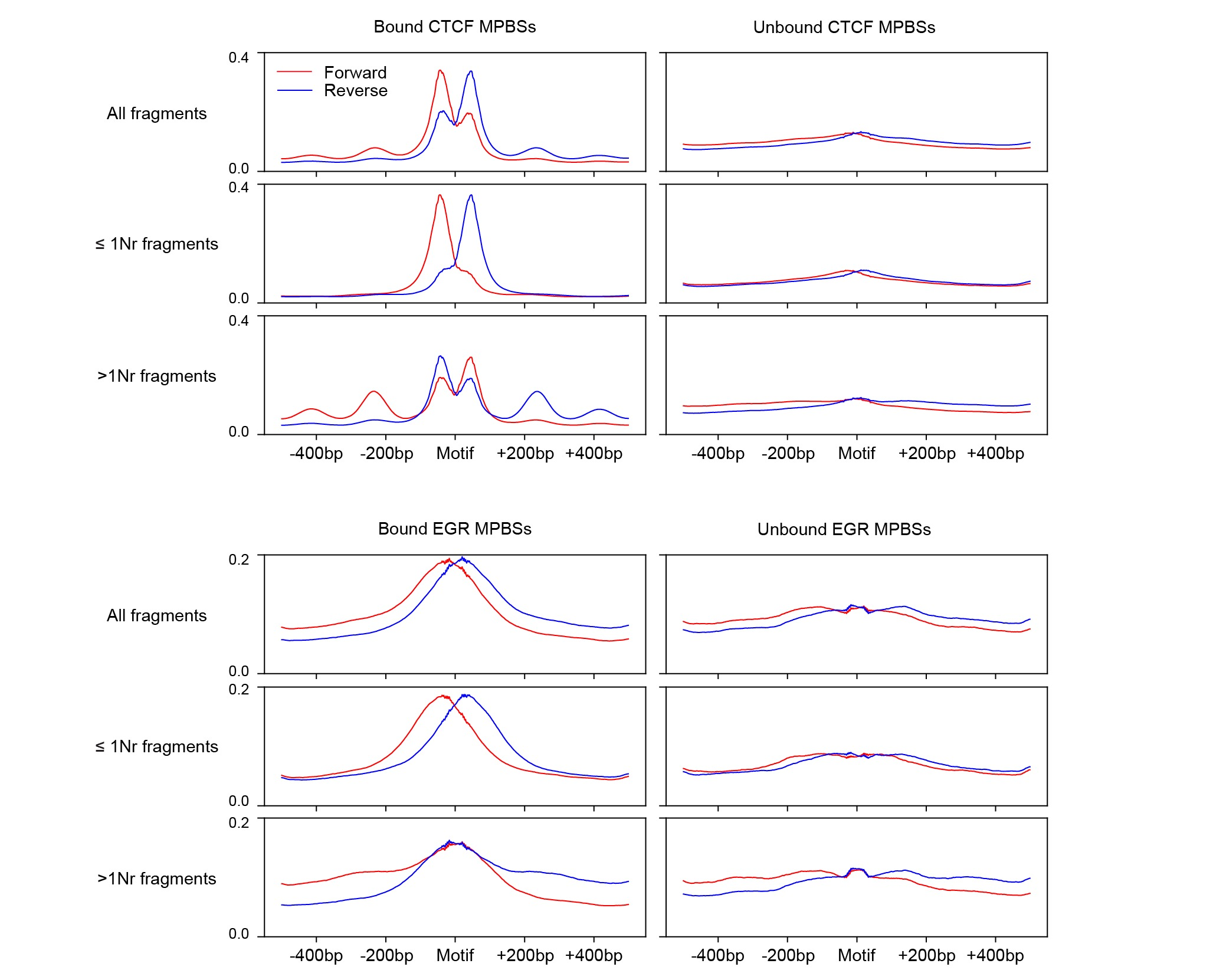

Supplement: S4 Fig — Cleavage signals were processed without manual bias correction. Three plots were made for each class of MPBSs using all ATAC-seq reads, ATAC-seq reads with maximum fragment size at 1Nr, and ATAC-seq reads with minimum fragment size more than 1Nr. Nr, nucleosome size. MPBS, motif-predicted binding site. (TIF) [file pcbi.1009921.s004.tif]

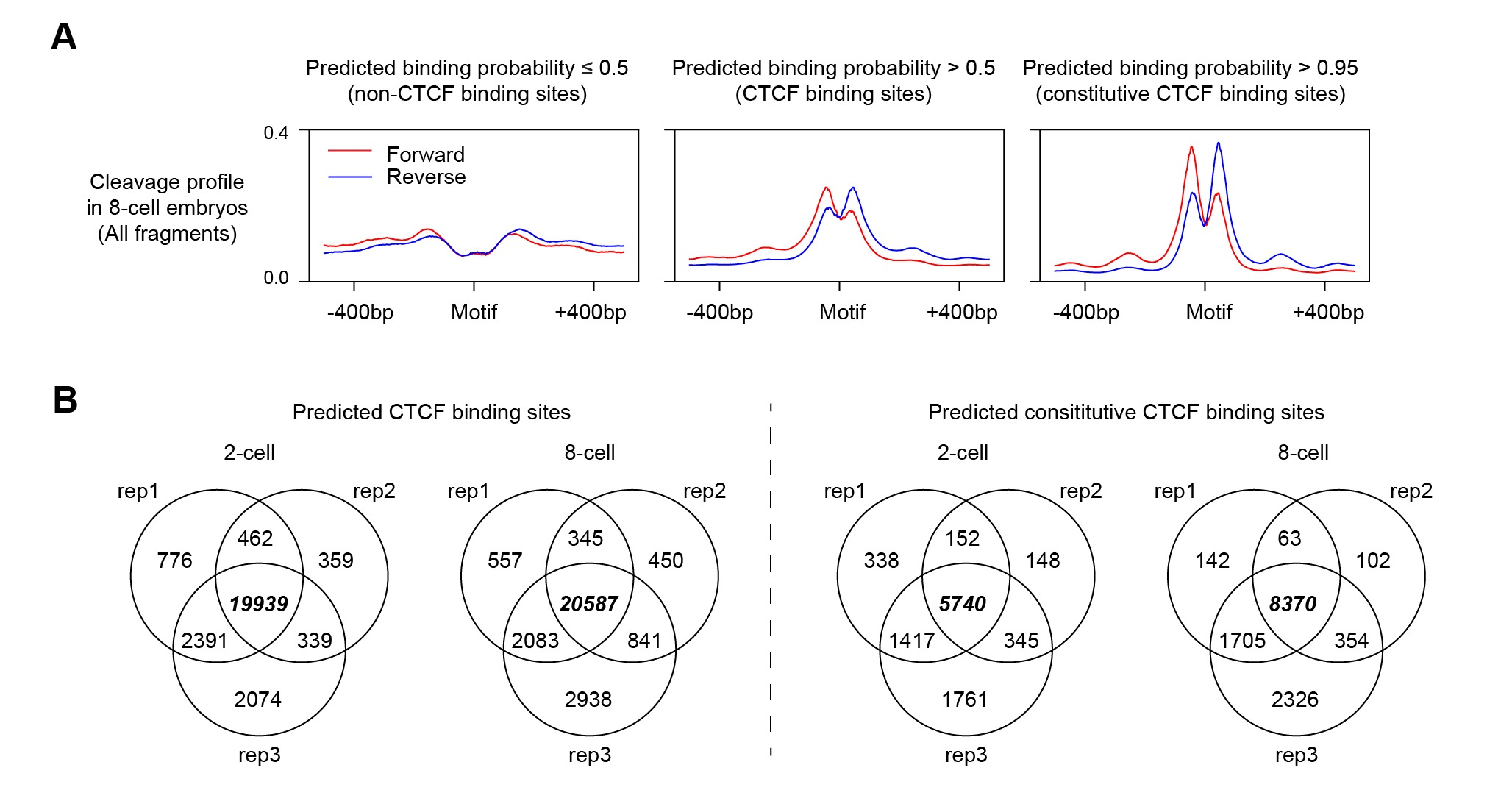

Supplement: S5 Fig — (A) Metagene plots show aggregated cleavage profiles at predicted unbound, bound, and constitutively bound CTCF MPBSs in 8-cell embryos. (B) Venn diagrams show the number of predicted bound/conductively bound CTCF MPBSs in 2-cell and 8-cell embryos from 3 experiments. CTCF binding sites predicted in all three experiments are regarded as true binding sites. (TIF) [file pcbi.1009921.s005.tif]

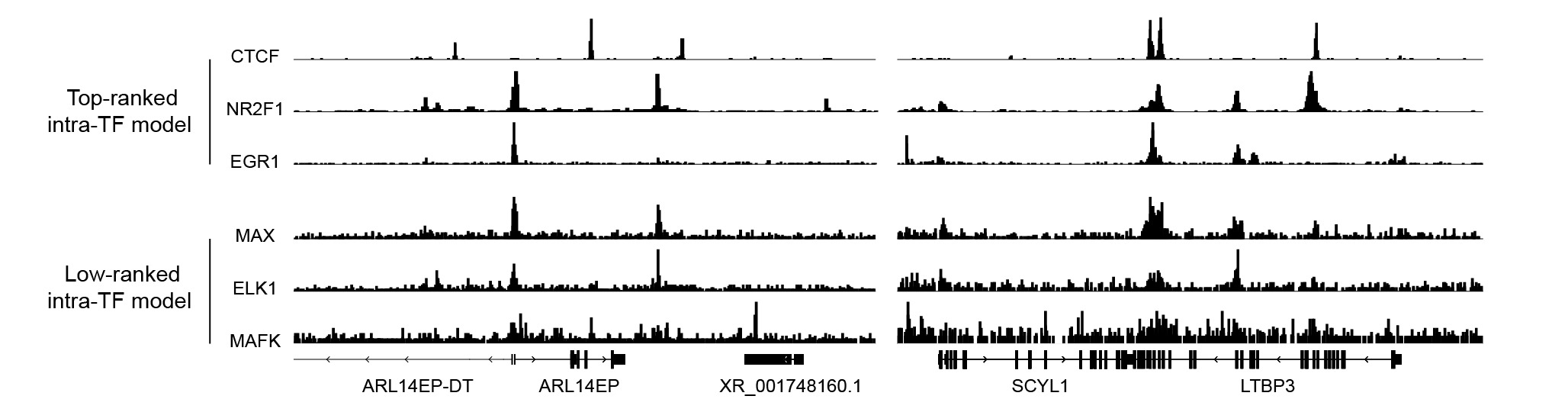

Supplement: S6 Fig — (TIF) [file pcbi.1009921.s006.tif]
