# Supplementary material for: TAMC: A deep-learning approach to predict motif-centric transcriptional factor binding activity based on ATAC-seq profile
Source: PLoS Comput Biol. 2022 Sep 12;18(9):e1009921. doi: 10.1371/journal.pcbi.1009921 (PMC9499209; doi:10.1371/journal.pcbi.1009921)
Supplement: Attachment — Submitted filename: Responses to review comments.docx [file pcbi.1009921.s013.docx]

Dear Editor,

We are pleased to hear that the reviewers found most of their questions have been answered to their satisfaction in the 1^st^ round of revision. In this 2^nd^ round of revision, we made corrections in wording and spelling according to the suggestions of reviewer 1. We also incorporated new data (Figure 1C, Figure S2A and Figure S6) to provide answers for the new comments from reviewer 2. All changes within the manuscript have been highlighted.

Please find our point-by-point replies to the reviewers below.

Yours sincerely,

The authors

**Responses to review comments:**

Reviewer #1: I find the manuscript greatly improved and the responses to all reviewers thorough and clear.

Two minor points:

* "including" implies a non-exhaustive list ("two representative footprinting tools including TOBIAS and HINT-ATAC"). I would suggest "namely", ie "two representative footprinting tools, namely, TOBIAS..."

Thank you for the suggestion. We replaced “including” to “namely” in the revised manuscript.

* "matric" (multiple places) should be "metric" I think. Eg, "Tobias applies the same matric...", "FPS matric" etc.

Thank you for pointing out this spelling mistake. We corrected "matric" to “metric” throughout the revised manuscript except for “position weight matrices (PWMs)”.

Reviewer #2: The authors addressed most of my concerns. The revised Figure 1A makes it much easier to understand the CNN structure used in this study. However, I still have some questions:

1) I am still trying to understand why the model requires longer input regions to achieve the best performance. I am wondering if there is a positional bias between bound and unbound MPBSs. For example, bound MPBSs may be more centrally positioned with respect to ATAC-seq peak summits, while unbound MPBSs are more peripheral, which may introduce systematic ATAC-seq signal bias. If this is the case, the difference in ATAC-seq read intensity in the 1kb region centered at MPBS may be enough to distinguish unbound MPBS from bound MPBS. Please demonstrate that there is no positional bias. If the bias exists, the authors should control it when making the balanced training set when subsampling the unbound MPBSs, and repeat the analysis

Based on this comment, we realize that our original Figure 1C might cause misunderstanding that unbound MPBSs are located at periphery of ATAC peaks. In order to provide a complete representation of unbound MPBS distribution, we changed Figure 1C to a new representative locus within FRMD48 gene. It is known that TF binding events typically take place at open chromatin regions that is characterized by high ATAC-seq read intensity, or high chromatin accessibility. Therefore, it is expected that the bound MPBSs mainly locate close to the summits of ATAC peaks. However, as shown in the revised Figure 1C, the unbound MPBSs could either locate at the periphery of ATAC peaks (the left unbound MPBS in revised Figure 1C) or close to the summits of ATAC peaks (the right unbound MPBS in revised Figure 1C). This suggests that high chromatin accessibility is a necessary but insufficient requirement for TF binding. Consequently, predicting bound MPBSs merely based on ATAC-seq read intensity will cause high false positive rate.

We also need to clarify that we used Tn5 cleavage signal (which is determined by +5/-4bp from the end of each ATAC-seq read), rather than ATAC-seq read intensity, to prepare input signal. ATAC-seq read intensity only provides a rough measurement of chromatin accessibility. In contrast, the Tn5 cleavage signal not only maps open chromatin regions (which is characterized by high Tn5 cleavage signal frequency), it also maps the precise TF binding sites within open chromatin regions (which is often shown as a footprint region centered around the MPBS in Tn5 cleavage profile). In addition, the pattern of footprint varies for different TFs. For example, TFs that bind in dimerization modes usually have wider footprint than TFs that bind in monomer. Furthermore, the binding of some TFs is accompanied with co-factors that interact with the chromatin at regions either close or distal to MPBSs. A MPBS located at regions with high chromatin accessibility but without correct footprint patten or corresponding co-factor interacting features at surrounding regions should not be a *bona fide* TF binding site.

In sum, the features of TF binding sites can be divided into two classes: 1) common binding features for all TFs (such as the high chromatin accessibility and appearance of footprint pattern); 2) unique binding features for a specific TF (such as the pattern of footprint and the surrounding co-factor binding features). While existing footprinting methods (HINT-ATAC and TOBIAS) only considered the common features, our method further reduces false positive rate by considering both common and unique binding features. Importantly, the longer input signal we use, the more information, especially the unique binding features, can be captured and utilized for prediction. This explains why longer input regions achieve better performance.

2) The raw AUROC for all experiments is quite helpful to better understand the results. As such, I would suggest that the authors directly compare AUROCs across different models using 2~3 representative TFs.

According to the suggestion, we added Figure S2A to show direct comparison of AUROCs obtained from three experiment replicates across different models using 4 representative TFs (CTCF, SRF, E2F4 and MFIC). Among these 4 TFs, TAMC give best prediction of CTCF and SRF binding sites for all testing ATAC-seq sequencing depth, while E2F4 and MFIC show better TAMC prediction than the other two methods only at low testing ATAC-seq sequencing depth. We also added a new spreadsheet in Table S3 listing direct comparison results of AUROCs for all 47 TFs as supplementary information for Figure S2A. The new spreadsheet also shows that the advantage of TAMC in TF prediction becomes more obvious as the sequencing depth of testing ATAC-seq data get lower. This is consistent with the conclusion obtained from our previous comparisons using AUROC ranks.

3) After a better understanding of “scaled AUC” in Figure 4, I find it quite interesting that ~30% of TFs achieve better performance when using models trained on other TFs. Although I agree with the authors that intra-TF prediction is generally better than cross-TF prediction for most TFs, I am also curious if there are any common properties shared by these “unusual” TFs.

We think this question raised by Review 2 is very interesting and important for further studies. Here we provided two properties shared by the “unique TFs”, and we also provided potential methods to improve intra-TF performance of these “unique TFs”.

1. We have noted that many “unusual” TFs have very low number of bound MPBSs within each cell type (e.g., ATF3, CEBPB, E2F4, NR2C2, RFX5, TBP, ZSCAN29) (Figure S1). This results in insufficient data amount for training the model to capture common and/or unique features precisely. In the discussion section, we have provided one possible way to solve the first problem consisting in combining labeled MPBSs for the same TFs from different cell types for training. We have also demonstrated that this solution can not only increase data amount but also prevent cell type-specific overfitting effect.

2. Compared to the TFs located at the top rows in Figure 4 heatmap (e.g., CTCF, NR2F1 and EGR1), we also noted that some “unique” TFs located at the bottom rows in Figure 4 heatmap (e.g., MAX, ELK1 and MAFK) have higher noise background in their ChIP-seq data (Figure S6). High noise background reduces the accuracy in MPBS labeling, and thus impairs the trained model’s ability to capture correct binding information.

High ChIP-seq noise is usually caused by poor specificity of the antibody used in ChIP experiment and cannot be solved by combining MPBSs from different cell types easily. As discussed in our responses for the first comment, the performance of TAMC models is a combinational reflection of the model’s capability in capturing both common and unique binding features. If a TF achieves similar or even better performance when using models trained on other TFs, it means that its binding site could be predicted by the common binding features in models trained on other TFs. Based on this logic, a potential solution is to separate the training into two steps: the first step uses MPBSs of all TFs to capture common binding features, while the second step applies transfer learning by continuing training the model using MPBSs of a specific TF to capture unique binding features. For TFs with high noise in their ChIP-seq data and show compromised performance after TF-specific training, we can predict their binding sites just based on the common binding features using the model obtained in the first step. We incorporated this part into the discussion section in the revised manuscript as a potential direction for future studies.

Reviewer #3: All of my questions have been answered to my satisfaction

We thank Reviewer 3 for his/her comments and feedback during the revision, and we are pleased that the Reviewer found our revision have addressed all his/her questions.
