# Supplementary material for: TAMC: A deep-learning approach to predict motif-centric transcriptional factor binding activity based on ATAC-seq profile
Source: PLoS Comput Biol. 2022 Sep 12;18(9):e1009921. doi: 10.1371/journal.pcbi.1009921 (PMC9499209; doi:10.1371/journal.pcbi.1009921)
Supplement: Attachment — Submitted filename: Response to reviewer comments.docx [file pcbi.1009921.s012.docx]

Dear Editor,

Thank you for the detailed report and the insightful comments from the reviewers.

We have modified the manuscript according to the recommendation and summarized the changes as listed below. We tracked all changes and highlighted the context related to the reviewers’ major questions in the marked-up copy of the revised manuscript.

Please find our point-by-point replies to the reviewers in the following pages.

Yours sincerely,

The authors

**Summary of changes:**

1. We modified the schematic of model and input structures in Figure 1 and the corresponding context to provide more details of the deep learning architecture.

2. We added error bars and indicated statistical significance (Friedman-Nemenyi test) for all line/bar graphs. The complete test results are provided in Table S3. To leave space for labeling significant differences, we reorganized original Figure 2B/C to new Figure 2B-D and S2A/B.

3. We provided a TF-to-TF heatmap using raw AUROC values (Figure S3) and explained why we need row scaling for the heatmap as shown in Figure 4.

4. We added new analyses to check how changing intercepts for fragment size separation (Figure 5C) and lengths of input signals (Figure 5D) influences the TAMC performance. We also provided example footprint/cleavage metagene profiles (Figure S4) and re-wrote the context related to Figure 5 to provide a better explanation of why TAMC can generate TF-specific models.

5. We added an example of applying TAMC to predicting CTCF binding sites using ATAC-seq data of human zygotes (Figure 6 and S6).

6. We modified Table 1 to clarify features of different methods, especially for the intra- and cross-data prediction settings.

7. We improved the wording and avoided using uncommon acronyms according to the reviewer’s suggestions.

8. We provided the raw AUROC values (Table S4) and raw binding probability predictions (Table S5) used to draw the conclusions in the manuscript.

9. Beyond the reviewers’ comments, we made two more corrections in the main figures:

1) We noted that our original HepG2 heatmap in Figure 2A might cause confusions about the intra-/cross-data settings of HINT-ATAC method. In the previously submitted manuscript, we referred it in the figure legends as intra-data prediction based on TAMC settings. However, for HINT-ATAC, the prediction should be cross-data as we used published HINT-ATAC models that are pre-trained on GM12878 ATAC-seq data. In the revised manuscript, we replaced this HepG2 heatmap with a heatmap using TAMC model trained on GM12878 ATAC-seq and K562 ATAC-seq data as testing data. In this way, the new Figure 1A could be representation for the distribution and degree of changes in both intra- and cross-data comparisons.

2) We found the order of variant 3 and 4 of TAMC input structures in the original heatmap of Figure 5B is reversed. We corrected that mistake in the revised manuscript.

**Reply to reviewers:**

Reviewer #1:

The paper describes an interesting deep-learning approach to predict transcription factor binding sites using motif information, ATAC-seq profiles, and ChIP-seq information. While it seems a worthwhile approach, I feel some aspects would benefit from greater clarity.

Thank you for the positive feedback on our method. We made modifications according to recommendation, and we agree that these modifications greatly improved the clarity of our manuscript.

Major:

Results, page 6, "genomic cleavage signals and slopes are generated following HINT-ATAC input signal processing strategy..." is the strategy reimplemented, or is HINT-ATAC used directly?

We reimplemented part of the HINT-ATAC strategy to calculate and normalize genomic cleavage signals and slopes. For making default TAMC input signals, we modified HINT-ATAC scripts to avoid doing bias correction. For making variant inputs for TAMC, we further modified HINT-ATAC signaling processing scripts to change size and strand separation criteria.

To prevent misunderstanding that HINT-ATAC is directly used for preparing cleavage profiles of TAMC input signals, we reworded the sentence related to default TAMC input processing to "…genomic cleavage signals and slopes are calculated and normalized using HINT-ATAC input signal processing scripts with modifications at the bias correction step". We also modified Figure 1A and 5A to avoid using “TOBIAS” and “HINT-ATAC” in schematics of input structures.

Page 7: "we compared TAMC predictions with predictions generated using previous footprinting tools including TOBIAS and HINT-ATAC". These are the only two previous tools compared. The sentence should be reworded.

We agree that the original sentence is misleading. We reworded the sentence to “…we compared TAMC predictions with predictions generated using two representative footprinting tools including TOBIAS and HINT-ATAC…".

Page 8, “compared to TOBIAS and HINT-ATAC, TAMC gave best cross-cell prediction…” however, table 1 suggests that the former two tools cannot be used for cross-data. This should be clarified.

We apologize for the confusion in the table related to prediction settings for different methods. In the resubmitted manuscript, we revised Table 1 to clarify the following features of the HINT-ATAC, TOBIAS and TAMC methods:

1. The TOBIAS method uses an FPS metric with fixed parameters and does not need model training. Therefore, there is no distinction between intra- and cross-data prediction for TOBIAS. In contrast, both HINT-ATAC and TAMC required model training, and can be used for TFBS prediction using the same (intra-data) or different (cross-data) testing ATAC-seq data as the training ATAC-seq data. In our study, we used the published HINT-ATAC models that is pre-trained on GM12878 ATAC-seq data for testing. Therefore, HINT-ATAC predictions on HepG2 and K562 ATAC-seq data could be regarded as cross-data predictions.

2. The original HINT-ATAC paper has reported that there is no difference in the HMM model trained based on binding sites of different TFs (1). Therefore, all HINT-ATAC perditions were made using the HMM model trained based on EGR1 binding sites. Similarly, as TOBIAS applies the same matric to calculate the footprint scores at all genomic sites, the predictions made by TOBIAS is not TF-specific as well (2). In contrast, our study showed that TAMC can tell TF-to-TF differences at their binding sites and generates TF-specific models to improve prediction accuracy (Figure 4 and 5). We missed this feature in the original Table 1 and add it in the revised manuscript.

3. In our study, we showed that TOBIAS gave much better prediction results than HINT-ATAC, no matter whether the HINT-ATAC prediction is performed in intra- or cross-data settings (Figure 2). However, the performance of TOBIAS is limited by the sequencing depth of testing ATAC-seq data. Compared to TOBIAS, our TAMC method showed better prediction performance in both intra- and cross-data settings as long as the training ATAC-seq data has higher sequencing depth than the testing ATAC-seq data (revised Figure 2D and Figure S2). In addition, the performance of TAMC can be further improved by increasing the sequencing depth of training ATAC-seq data, which is easily accessible from public dataset (revised Figure 2C). Therefore, we concluded that the performance of TAMC is better than HINT-ATAC and TOBIAS in general. We used +++, ++ and + symbols to represent high, mid and low evaluation of performance of different methods in the table. In addition, we missed the feature related to sequencing depth in original Table 1 and we added it in our revised manuscript.

Page 12, figure 4: Why are the AUC values scaled between -1 and 1? This seems an unusual procedure and makes it impossible to compare rows. Why not plot the raw AUC on a 0-1 scale as usual? This should be clarified.

The aim of Figure 4 is to investigate whether the TAMC prediction is TF-specific or not. In the heatmap, we listed testing TFs in rows and training TFs in columns. For each testing TF, we need to compare performance of models trained on different TFs (cross-TF) with models trained on the same TF as testing TF (intra-TF). This requires us to compare columns of each row in the heatmap.

In our revised manuscript, we added a heatmap plotted using the raw AUROC values as shown in Figure S3. Because raw AUROC values are very close to each other within each row, it very difficult to tell the difference between columns. Therefore, to make the column differences more obvious, we did row scaling in Figure 4.

In the resubmitted manuscript, we provided an equation in figure legends to describe how the row scaling is performed:

scaled AUROC = log_2_(AUROC/intra-TF AUROC)

briefly, we first calculated the ratios between AUROC values of TAMC models trained on 47 TFs and the AUROC value of intra-TF TAMC model in each row; then we normalized the ratios by log_2_ transformation. Majority of the obtained scaled AUROC values falls within the range from -0.1 to 0.1. A positive scaled AUROC values means better cross-TF predictions than intra-TF predictions, while negative scaled AUROC values means better intra-TF predictions than cross-TF predictions. As most scaled AUROC values in the heatmap are negative, we concluded that the optimal TAMC prediction should be obtained under intra-TF settings. In other word, TAMC can tell TF-to-TF differences at their binding sites and generate TF-specific models to improve prediction accuracy.

General: a bit more detail in the description of the deep learning architecture would be desirable.

Thanks for the suggestion. We revised Figure 1A and provided more details about the deep-learning architecture in the context of the first paragraph of TAMC overview.

General: It would be good to include error bars wherever possible, ie figures 2b, 2c, 3.

Thanks for the suggestion. We added error bars and provided statistic significances calculated by Friedman-Nemenyi test. In the figures, we indicated significant difference between TAMC and other models, or between default TAMC model with variant TAMC models. We also provided complete significance test results for all comparison in Table S3.

Minor:
Abstract: “TAMC captures both footprint and non-footprint features…” do the authors mean “utilizes” (not “captures”)?

TAMC captures both footprint and non-footprint features during training, and then utilizes both two types of features for making predictions. To avoid misunderstanding, we reworded the sentence to “TAMC make predictions based on both footprint and non-footprint features…” in the revised manuscript.

Author summary: avoid acronyms here, like CNN, TFBS, ATAC-seq, or explain them, since this is for non-specialists

Thanks for the suggestion. We avoided using any of these acronyms in the revised summary part.

Reviewer #2:

In this paper, the authors introduced a new computational method, TAMC, predicting TF-binding activity in open chromatin regions from ATAC-seq data. The authors showed that TAMC outperformed two existing methods, TOBIAS and HINT-ATAC, by combining them using a deep-learning approach without the correction of sequence bias on cut sites (e.g., Tn5 sequence preference). However, it is unclear how the convolutional network structure of TAMC is configured to train the dependency of local TF footprints and cleavage signals reflecting TF-binding biochemistry. It is also unclear whether TAMC can be generalized to other data sets. These points are discussed in more detail below:

Thank you for the constructive advice on our manuscript. According to recommendation, we paid special attention to clarification of TAMC deep-learning architecture and explanation of how TAMC learns the biology of TF binding sites when revising our manuscript.

Major points:

1) The convolutional layer architecture of TMAC shown in Figure 1A does not seem to integrate neighboring base-pair signals within a specific feature. Instead, it aggregates signals across neighboring features at the same position. In other words, the current architecture seems to randomly convolute footprinting and cleavage site features, which are then max-pooled without considering neighboring base pair signals. Since the order of the features is arbitrary, I am struggling to understand how the convolutional layer learns the biology of transcriptional binding sites. What do three different convolutional layers with kernel sizes (k=3, 5, 7) and color (red, green, blue) mean from a biological perspective?

Based on these questions, we realized that Figure 1A in the previously submitted manuscript may cause confusions related to the convolution and max-pooling steps. Therefore, in the resubmitted manuscript, we revised Figure 1A and provided more details about the deep-learning architecture in the context, especially for the convolution and max-pooling steps. We clarified that instead of extracting footprint and cleavage features randomly, we performed convolution with kernels moving at 1bp per step in the direction from -500bp to +500bp from the center of each motif predicted binding site. The convolution kernels could be regarded as scanners for TF binding sites. As the length of binding sites varies between TFs, we combined kernels with different sizes (k=3, 5, 7) to extract features for small and large binding sites. For each kernel size, we repeated convolution for 3 times to generated 3 feature maps. Therefore, in total 9 (3x3) feature maps were generated. The later max-pooling step was performed between the 3 feature maps from the kernel of the same size, and the aim of the max-pooling step is to generate a feature map that have the maximum feature signal at each bp. The red, green, and blue colors were used to mark feature maps originated from kernels with k=3, k=5, and k=7, respectively.

2) It is also unclear why such a long region (1,000 bp) is needed for modeling. A footprinting event is local and can be captured within 50bp for almost all TFs. Is it really required to have 1,000bp to achieve high accuracy? What happens if it is reduced to 500bp or even 100bp? What is the optimal length?

The chromatin cleavage patterns surrounding the footprint region are different between TFs. To show such difference, we added example metagene cleavage profiles surrounding binding sites of CTCF and EGR1 in our resubmitted manuscript (Figure S4).

In our revised manuscript, we then compared models based on 1000bp, 500bp and 100bp input regions, and we detected significant reduction in prediction performance as the modeling region shortens (Revised Figure 5D). Therefore, longer modeling region provides more cleavage information that are helpful for TFBS prediction. We conjected that the motif-distal cleavage features could reflect the variability in recruitment of co-factors for different TFs.

Considering most of ATAC peak regions are within 1000bp, increasing the length of modeling region to over 1000bp will not add much cleavage information that can be used for prediction. Therefore, we chose 1000bp as the optimal modeling length, which is the same as modeling length used in HINT-ATAC method.

3) When training and testing the model of TAMC, the authors used only three cell lines (GM12878, HepG2, and K562), for which many ChIP-seq datasets are available in the ENCODE database. However, no such ChIP-seq data sets are available for most human tissues, and it is unclear if the model can be extended to these human tissues. The authors need to demonstrate the generalizability of the model by analyzing bulk/single-cell ATAC-seq data sets from human tissues.

Our study has shown that our TAMC outperforms TOBIAS and HINT-ATAC no matter whether the training and testing ATAC-seq data are obtained from the same (intra-data) or different (cross-data) cell lines (Figure 2). In the resubmitted manuscript, we replaced the right heatmap in Figure 2A with a new heatmap showing cross-data AUROC values for GM12878 trained TAMC on K562 testing ATAC-seq data. The total number of aligned reads for the K562 testing ATAC-seq data shown in the new heatmap has been downsized to 25 million. This sequencing depth falls into the range (20-50 million aligned reads) that we normally obtained in real studies. Majority of the 47 TFs have good cross-data prediction AUROC values over 0.8 (highlighted in red in the tables). This explains the generalizability of the TAMC model to real research data. As GM12878, HepG2 and K562 are all human cell lines, we considered that the TAMC models trained using GM12878 and HepG2 ATAC-seq data could be used to predict TFBSs using ATAC-seq data of human tissues as well.

In our resubmitted manuscript, we added an example for applying TAMC to predict CTCF binding sites during zygotic genome activation (ZGA) of early human zygotes (Figure 6). Because our model uses sigmoid activation to generate probability of the two classes of binding status, we use 0.5 as the intercept to separate bound and unbound sited. We predicted sites with binding probability > 0.5 as CTCF binding sties, while sites with binding probability > 0.95 as constitutive CTCF binding sites. As expected, we only detected footprint patten in metagene cleavage profiles at predicted CTCF binding sites but not at the predicted non-CTCF binding sites (Figure S5A). In addition, the predicted constitutive CTCF binding sites exhibited deep footprint patten as a result of frequent or persistent TF binding events (24) (Figure S5A). Studies have shown key roles of CTCF in establishing chromatin structure during ZGA, and the expression of CTCF is drastically increased during ZGA (3). Our prediction showed that chromatin structure reorganization during ZGA is associated with both increase in the number and changes in the location of constitutive CTCF binding sites (Figure 6). This prediction is in consistent with known function of constitutive CTCF binding in maintaining 3D chromatin structures (4). All these information supported successful prediction of CTCF brining sites in human embryonic cells with TAMC. Therefore, although few ChIP-seq data sets are available for most human tissues, the performance of TAMC can be examined based indirect evidence.

4) In Figure 5B, the authors primarily used CTCF and EGR to demonstrate that TAMC can capture footprint features of TF-binding sites. However, a more systematic analysis is required to thoroughly test if the differential prediction performance across different model structures is specific to TFs. For example, the expression and the size of TFs could be associated with differences in the prediction performance across the model variants. This can potentially be used to improve the prediction performance further.

First, we need to apologize for a mistake in our original Figure 5B. We mistakenly reversed the order of TAMC variant 3 and 4 in the heatmap. This might cause confusions for the reviewers to understand the figure. We corrected this mistake in our revised manuscript. The revised Figure 5B showed that removing strand information (variant 3) affected binding site prediction for CTCF but not for EGR1, while removing fragment size information (variant 4) affected binding site prediction for EGR1 but not for CTCF. Therefore, strand and fragment size information are TF-specific requirement for making better predictions for CTCF and EGR1, respectively. This is in agree with the reviewer’s comment that “the differential prediction performance across different model structures is specific to TFs”.

Second, to provide deeper investigation of how strand and/or fragment size information helps to predict TF brining sites, we compared metagene cleavage profiles at labeled CTCF and EGR1 bound sites (Figure S4). We demonstrated that the strand information in TAMC inputs might help to capture the strand-specific cleavage pattern at CTCF binding sites, while the fragments size separation in TAMC inputs helps to capture the small footprint pattern at EGR1 binding sites that is only revealed by cleavage profiles generated by small reads fragments. In other words, a certain TAMC input structure can help to improve binding sites prediction for specific factors through facilitating recognition of their unique cleavage features.

Third, in the revised manuscript, we added discussion about interpreting the TF-specific cleavage features from the aspect of TF binding biology. The strand-specific cleavage pattern around CTCF binding site might reflect its dimerized binding mechanisms, while the shallow footprint pattern at EGR1 binding sites could reflect its transient activity at most of its binding sites. Therefore, we can improve TAMC performance for specific TFs by modifying input structure based on the biological mechanisms behind their binding events.

Finally, the heatmap of Figure 5B has provided a systematic analysis of which TFs are benefited from a specific TAMC model in their binding site prediction. As we have shown with the example of CTCF and EGR1, thoroughly interpretation of the models’ differential prediction performance will provide instructive information to improve the prediction performance for a specific TF further. However, we hope the reviewers can agree that these analyses require a lot of work to carefully examine cleavage profiles of each TF with the knowledge of their biological binding mechanisms and, are therefore out of the scope of this manuscript which is mainly focused on providing a more general method for all TFs.

5) The authors showed that TAMC achieved robust performance regardless of the bias correction, implying that the TAMC model also learned features associated with bias and corrected them internally. If this is the case, I expect the prediction results to be concordant between the bias-corrected and uncorrected inputs. Please demonstrate this.

In Figure 3, we have shown that TAMC prediction results are concordant between the bias-corrected and uncorrected inputs by comparing the performance of TAMC using default inputs (completely bias uncorrected inputs) with TAMC using inputs with different combinations of bias-corrected footprint and/or cleavage profiles. In our revised manuscript, we added statistic test results for these comparisons to provide stronger evidence for the conclusion. As the reviewer mentioned, this implies that TAMC models were trained to correct Tn5 cutting bias internally. We incorporated this implication in the context of our revised manuscript.

6) Direct and quantitative comparison of AUC scores is required throughout the manuscript. In Figure 2A, it is unclear how significantly AUCs are different between TAMC and TOBIAS/HINT-ATAC. The distribution of AUC scores should be compared among the three methods, and the statistical significance of the differences should be assessed. The average AUC rank is not a good metric, either. Please consider it to something more direct in Figures 2B/C, 3, and 5B. In Figure 4, what is the normalization method to scale AUC (-0.1~0.1) by row? It is also unclear why the AUC should be scaled in this analysis.

We use AUROC ranks because we are more interested in detected the direction of change rather than the degree of change in performance. Similar ranked comparison has implemented by several previous footprint tool publication, including the original HINT-ATAC paper (1). At the same time, the distribution and degree of changes in AUROC values could be still observed from the representative heatmap (Figure 2A).

In the revised manuscript, we provided more details about how the average AUROC rank is calculated in the Method part: first, we ranked the AUROC values for each TF generated by different methods/models in each figure panel; second, we calculated the mean AUROC rank for each method/model per TF obtained from three repeated experiments; finally, we averaged the mean AUROC ranks of each method/model for all 47 TFs for comparison. To show the distribution of AUROC ranks for different TFs, we added error bars (which represent for standard error of mean) in all figures. We also assessed statistical significance using Friedman-Nemenyi test and provided p-values in the figures and in Table S4.

The aim of Figure 4 is to investigate whether the trained 1D-CNN models used for TAMC prediction is TF-specific or not. For a specific testing TF, we compared prediction performance of models trained using other TFs (cross-TF) with models trained on the same TF as testing TF (intra-TF). This requires us to compare columns of each row in the heatmap. As shown in Figure S3 in our revised manuscript, the raw AUROC values are very close to each other within each row. To make it easier to tell the difference between columns in the heatmap, we did row scaling in Figure 4. In the resubmitted manuscript, we provided an equation in figure legends to describe how the row scaling is performed:

scaled AUROC = log_2_(AUROC/intra-TF AUROC)

Briefly, we first calculated the ratios between AUROC values of TAMC models trained on 47 TFs and the AUROC value of intra-TF TAMC model in each row, and then normalized the ratios by log_2_ transformation.

Minor points:

1) AUC is the abbreviation of Area Under Curve, and it is not ROC-curve specific. Please mention this is the AUC of ROC in the method section.

Thanks for the suggestion. We changed “AUC” to “AUROC” as abbreviations for Aera Under ROC-curve in the revised manuscript.

2) MPBS is not a commonly used acronym. Please consider not using it.

Thanks for the suggestion. We agree that MPBS is not a commonly used acronym. However, as our method is specifically designed to predict TF binding status at sites that are first predicted by their binding sequencing motifs, we need to mention this definition very frequently in the manuscript. To avoid using the long definition phrase constantly, we used the abbreviation motif-predicted binding site (MPBS) following the published HINT-ATAC paper (Li *et al*., 2019).

In our revised manuscript, we made the following modifications to reduce difficulties for readers to understand this abbreviation:

1. We provided a complete explanation of MPBS at its first appearance in the Introduction part: “TAMC takes a combination of footprint profile and genomic cleavage profile (signals and slopes) around 500bp from the center of potential TF binding sites predicted by their binding sequence motifs, or motif-predicted binding sites (MPBSs), as input signal.”

2. We explained MPBS in the figure legend every time when it is used in the figures.

3. We reduce the frequency of using “MPBS” in the context when it can be replaced by other words.

3) Typos:

• “stand” in line 12 of page 13 should be “strand.”

• “sing” in line 6 of page 19 should be “using.”

Thanks for pointing out the typos. We corrected them in the revised manuscript.

Reviewer #3:

This paper proposes a CNN model to predict TF binding site (TFBS) using footprint and non-footprint (cleavage signal and slope) features derived from ATAC-seq data. Their TF-specific CNN model aggregates the footprint scores at base resolution and cleavage profiles using 1-D convolutional filters in the region (1000 bps) around the center of the motif predicted binding sites. It is shown that the model performance is independent of correcting for the sequence-specific cleavage bias present in the ATAC-seq data; a property that does not exist in the other tools for TFBS prediction using ATAC-seq profiles.

I have the following questions,

Thank you for highlighting one of the most important features of our method. We provided answers for all your question, and also incorporated these answers into our revised manuscript.

1) Please explain why the cutoff of 1 nucleosome size was used for the ATAC-seq reads (Figure 1B)? Also, explain why adding more intervals for the read size might be redundant, e.g., < 1Nr, 1Nr < - < 3 Nr, >3Nr, given that the inclusion of read size information affects the performance (Figure 5B)?

The cleavage profile part of TAMC input is processed following similar steps as HINT-ATAC. HINT-ATAC uses 1 nucleosome size as default cutoff for its HMM model input as it gives the best prediction. We took the same cutoff as the default signal processing strategy in TAMC.

In Figure 5B, we have shown that using input without separating read fragment sizes did not cause significant overall changes in TAMC performance (default vs. variant 4). In our resubmitted manuscript, we further showed that adding one more interval (1Nr and 2Nr) does not change overall TAMC performance as well (Figure 5C). We did not add another interval (3Nr) as majority of ATAC-seq read fragments are within 3Nr length. These results suggest that unlike HINT-ATAC, the overall performance of TAMC is not affected by read fragment sizes. However, for certain TFs, separating reads by fragment size improves prediction results. In other words, if TAMC is used for predicting binding site of a specific TF, its performance can be optimized by adjusting sizes of fragment reads used for prediction.

2) Please explain the choice of K562 cell line as the target dataset and GM12878 and HepG2 as the source datasets in the cross-data experiments (Figure 2B,C).

Our study requires comparing the performance of TAMC with HINT-ATAC and TOBIAS. While TOBIAS does not require model training, the published HINT-ATAC models are trained using GM12878 ATAC-seq data and has been tested for cross-data performance using K562 ATAC-seq data. Therefore, in our study, we used the same cell lines for training and testing TAMC.

Our study also requires another training cell line to investigate the performance TAMC models trained on multiple cell lines. We chose HepG2 cells because of the accessibility of deeply sequenced ATAC-seq data of this cell line in ENCODE data base. In addition, the ENCODE database provides HepG2 ChIP-seq data sets of many TFs that overlaps with GM12878 and K562 cell ChIP-seq data sets. This allows us to do cross-data analysis using TAMC models trained on multiple ATAC-seq data.

3) In Figure 2C, which dataset(s) were used as the source dataset for TOBIAS and HINT-ATAC?

The TOBIAS method uses an FPS metric with fixed parameters and does not need model training. For HINT-ATAC, we used the published HINT-ATAC models that are trained using GM12878 ATAC-seq data for testing. We added this information in related context in our revised manuscript.

4) Average AUC rank over the TFs is used to compare the performance of different models or training settings. Please provide a way for testing/explaining the significance of delta X in average AUC rank, i.e., is a decay of 0.1 in average AUC rank significant?

In the revised manuscript, we provided more details about how the average AUROC rank is calculated in the Method part: first, we ranked the AUROC values for each TF generated by different methods/models in each figure panel; second, we calculated the mean AUROC rank for each method/model per TF obtained from three repeated experiments; finally, we averaged the mean AUROC ranks of each method/model of all 47 TFs for comparison.

To show the distribution of AUROC ranks for different TFs, we added error bars in all figures. We assessed statistical significance using Friedman-Nemenyi test and indicated significant differences in all figures. We also provided the complete statistic test results for all figures in Table S3.

**Reference**

1.	Li ZJ, Schulz MH, Look T, Begemann M, Zenke M, Costa IG. Identification of transcription factor binding sites using ATAC-seq. Genome Biol. 2019;20.

2.	Bentsen M, Goymann P, Schultheis H, Klee K, Petrova A, Wiegandt R, et al. ATAC-seq footprinting unravels kinetics of transcription factor binding during zygotic genome activation. Nat Commun. 2020;11(1).

3.	Chen XP, Ke YW, Wu KL, Zhao H, Sun YY, Gao L, et al. Key role for CTCF in establishing chromatin structure in human embryos. Nature. 2019;576(7786):306-+.

4.	Khoury A, Achinger-Kawecka J, Bert SA, Smith GC, French HJ, Luu PL, et al. Constitutively bound CTCF sites maintain 3D chromatin architecture and long-range epigenetically regulated domains. Nat Commun. 2020;11(1).
